# Supplementary material for: Phosphorylation toggles the SARS-CoV-2 nucleocapsid protein between two membrane-associated condensate states
Source: Nat Commun. 2025 Aug 26;16:7970. doi: 10.1038/s41467-025-62922-4 (PMC12381204; doi:10.1038/s41467-025-62922-4)
Supplement: Supplementary file 2 — Description of Additional Supplementary Files [file 41467_2025_62922_MOESM2_ESM.pdf]

Description of Additional Supplementary Files

File Name: Supplementary Movie 1

Description: N + 1-1000 RNA + GUVs with no membrane protein fragment. Widefield imaging sequence of several condensates composed of N protein + 1-1000 RNA being held by optical tweezers surrounding a GUV located at the center of the field of view. One condensate is brought to the surface of the GUV and then pulled away repeatedly without causing deformation of the condensate or GUV. Video taken at 2 frames per second. Video related to Fig. 2d.

File Name: Supplementary Movie 2

Description: pN + 1-1000 RNA + GUVs with no membrane protein fragment. Widefield imaging sequence of several condensates composed of pN protein + 1-1000 RNA being held by optical tweezers surrounding a GUV located at the center of the field of view. Several condensates are brought to the surface of the GUV and then pulled away without deforming the condensates or GUV. Condensates can also be held at the surface of the GUV for several seconds causing no deformation of the condensate, after which they can be pulled away from the surface. Video taken at 2 frames per second. Video related to Fig. 2d.

File Name: Supplementary Movie 3

Description: N + 1-1000 RNA + GUVs with $Nsp3^{1\text{-}111}$ membrane protein. Widefield imaging sequence of two condensates composed of N protein + 1-1000 RNA being held by optical tweezers and brought to the surface of a GUV coated with $Nsp3^{1\text{-}111}$ at the center of the field of view. The condensates attach to the surface. The larger condensate falls from the focal plane after being released by the tweezer, due to gravity. Video taken at 2 frames per second. Video related to Fig. 2d.

File Name: Supplementary Movie 4

Description: pN + 1-1000 RNA + GUVs with $Nsp3^{1\text{-}111}$ membrane protein. Widefield imaging sequence of several condensates composed of pN protein + 1-1000 RNA being held by optical tweezers and brought to the surface of a GUV coated with $Nsp3^{1\text{-}111}$ at the center of the field of view. The condensates wet the surface of the GUV and form a layer of protein over time. Video taken at 2 frames per second. Video related to Fig. 2d.

File Name: Supplementary Movie 5

Description: N + 1-1000 RNA + GUVs with $M^{104\text{-}222}$ membrane protein. Widefield imaging sequence of several condensates composed of N protein + 1-1000 RNA being held by an optical tweezer and brought to the surface of a GUV coated with $M^{104\text{-}222}$ at the center of the field of view. The condensates attach to the surface. One condensate is wrapped by the GUV

membrane over time, resulting in partial engulfment of the condensate. Video taken at 2 frames per second. Video related to Fig. 2d-e.

File Name: Supplementary Movie 6

Description: pN + 1-1000 RNA + GUVs with $M^{104\text{-}222}$ membrane protein. Widefield imaging sequence of several condensates composed of pN protein + 1-1000 RNA being held by optical tweezers surrounding a GUV coated with $M^{104\text{-}222}$ located at the center of the field of view. Several condensates are brought to the surface of the GUV and then pulled away without deforming the condensates or GUV. Condensates are held at the surface of the GUV for several seconds where they can fuse with other condensates and still be pulled away from the GUV surface, causing no deformation of the condensate or GUV surface. Video taken at 2 frames per second. Video related to Fig. 2d.
